# Neural circuits for mammalian parental behaviour

**DOI:** 10.1038/s41583-026-01073-x

**Published:** 2026-08-07

**Authors:** Bradley B. Jamieson, Johannes Kohl

**Affiliations:** State-dependent Neural Processing Laboratory, The Francis Crick Institute, London, UK

## Abstract

Most mammalian offspring are born helpless and depend on sustained parental care for survival, making caregiving one of the most conserved and essential behavioural repertoires in mammals. Yet infant-directed behaviours can be remarkably variable, ranging from care to neglect and aggression, even within the same individual. How neural circuits support both the robustness and flexibility of parental behaviour remains poorly understood. Here, we review recent advances in our understanding of the neural mechanisms of mammalian caregiving, placing circuit-level insights from rodent models into the broader context of parental diversity across mammals. Emerging evidence indicates that internal state and social experience dynamically reshape parental circuits across timescales from hours to weeks, and that these circuits adapt their function to generate life stage-appropriate behavioural output. Rather than acting as fixed control systems for instinctive actions, parental circuits are highly plastic and context dependent. Parental behaviour therefore provides a powerful model for understanding how neural circuits are reconfigured to meet changing behavioural demands.

## Introduction

Parental care is a central feature of mammalian reproduction. Compared with other vertebrates, mammals typically produce fewer offspring but invest heavily in their survival, a strategy that is particularly pronounced in species with helpless (altricial) young, such as humans, where offspring depend on prolonged caregiving^1–3^. Parental behaviour comprises a stereotyped repertoire of actions. In rodents, these include retrieval of pups to the nest, grooming and nursing, as well as auxiliary behaviours such as nest building. These behaviours are expressed with remarkable consistency across individuals.

A large body of work has established that parental behaviour is mediated by dedicated neural circuits whose organisation is increasingly well understood as a result of decades of work in laboratory mice (*Mus musculus*), rats (*Rattus norvegicus*), sheep (*Ovis aries*) and biparental voles (*Microtus ochrogaster*)^4–6^. Convergent evidence from these models supports the idea that there is a distributed network for parenting, spanning the extended amygdala systems involved in sensory and **valence** processing, hypothalamic hubs that create instinctive drive states, midbrain circuits for reward and action selection and cortical regions that contribute to learning and behavioural flexibility. In parallel, work in non-mammalian model systems, such as poison frogs and cichlids, has begun to reveal conserved principles of parental control across vertebrate classes^7–10^.

Despite the apparent stereotypy of parental behaviour, a central challenge remains: parental responses to offspring are highly variable, ranging from caregiving to neglect or aggression, even within the same individual^5,11–14^. This variability reflects a strong dependence of these behaviours on social experience, sensory context and **internal state**. Hormonal and physiological changes across timescales from hours to weeks profoundly alter parental circuit function, while prior social experience can similarly induce plasticity and alter behavioural output^5,11,15–18^. Recent studies have started to uncover the cellular and circuit-level mechanisms through which these factors modulate specific components of the parental network. It is also becoming clearer how parental circuits are reconfigured throughout life. For example, a recent preprint has reported that the functional circuits supporting parental behaviour emerge earlier in development than previously appreciated, via rapid, microglia-dependent neuronal remodelling^19^. Together, these findings point to a high degree of plasticity in circuits traditionally viewed as hardwired and restricted to adulthood.

In this Review, we synthesise recent advances in understanding the neural basis of parental behaviour, with a focus on rodents, where most mechanistic insights have been gained. Unless otherwise stated, the discussion refers primarily to uniparental rodent species, in which maternal care predominates. Where relevant, we explicitly distinguish findings from biparental species, in which paternal care has been studied extensively. We highlight how internal state, social experience and developmental stage shape parental circuits in males and females, and discuss interactions of parental behaviour with competing motivational drives. Finally, we outline key outstanding questions and future directions for the field.

## Diversity of mammalian parenting

Parental care is favoured when investment in current offspring increases lifetime fitness more than alternative uses of time and energy^2^. In mammals, gestation and lactation create a strong asymmetry in this investment between males and females, with exclusive maternal care being the most common pattern across species^2,3^. In contrast, males contribute to parenting in only 3–5% of mammals^20,21^. Parental care by non-parents (**alloparenting**) is common, occurring in 30–50% of species. However, regular or obligate alloparenting is largely confined to cooperatively breeding species, which comprise only 10–20% of mammals^22–24^. In general, whether care is provided by mothers, fathers and/or helpers is determined by a species’ ecological constraints and mating system^3,25^ (**Box 1**).

Who provides care is a useful way to organise parental diversity. In uniparental systems, caregiving is largely restricted to mothers and tightly coupled to **reproductive state**^2,25^. By contrast, in biparental and cooperative systems infant-directed behaviours can emerge outside pregnancy and **parturition** — in fathers, pair-bonded partners or non-breeding helpers — through social context and experience (**Box 1**). Moreover, in several biparental mammals, behavioural and endocrine changes associated with fatherhood emerge during the mate’s pregnancy, indicating that the transition into paternal care can begin before offspring are born^26^. **Cooperative breeding** represents a specialised form of alloparenting in which offspring are routinely cared for by both parents and additional group members (often non-breeding helpers), illustrating that parental behaviours can be expressed independently of biological parenthood^3,27,28^.

This diversity of caregiving strategies places important constraints on how parental behaviour is organised in the brain. If similar behaviours can be expressed by mothers, fathers and non-parental individuals, it is unlikely that each caregiving role relies on a wholly distinct circuit architecture. Rather, the occurrence of caregiving across parents and non-parents supports the existence of a shared neural substrate for parenting. Variation in who provides care is therefore more plausibly explained by differences in how a shared parenting circuit is recruited and modulated across physiological and social contexts than by its presence or absence. We next outline the organisation of this **core parental circuit**.

## Core parental circuitry

**Fig. 1:**
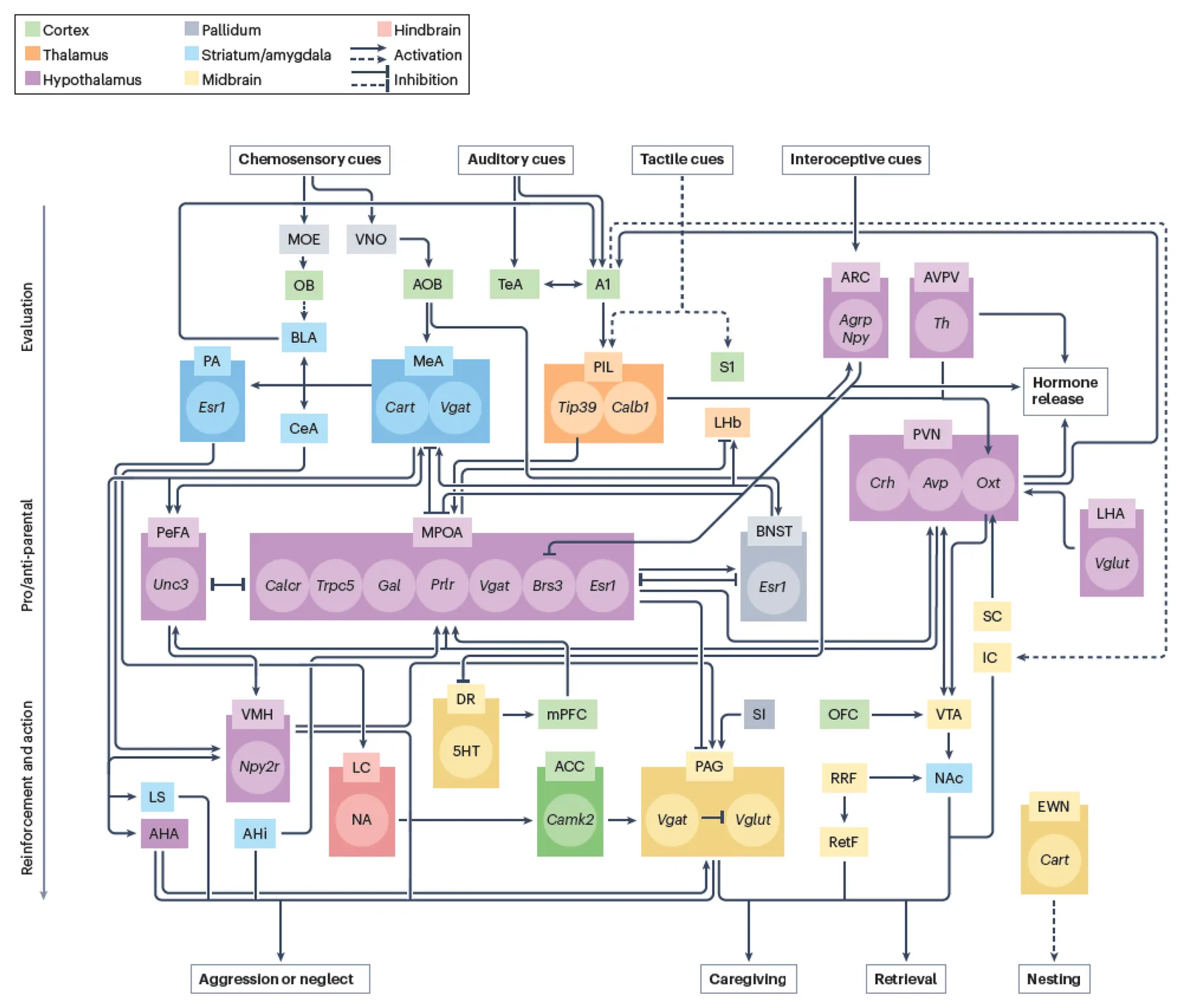
Functional circuit logic underlying rodent infant-directed behaviours. In rodents, infant-directed behaviour is controlled by distributed circuits that integrate pup-derived sensory cues with reproductive, hormonal and motivational state. Chemosensory, auditory and tactile signals are processed and evaluated through distinct sensory pathways^32,34,36,38,52,53,63,68^ and converge with endocrine and interoceptive signals onto hypothalamic and extended amygdala brain regions, including the medial preoptic area (MPOA) of the hypothalamus, a major hub for pro-parental behaviour^4,5,47,53^. These regions are thought to establish pro-parental and anti-parental states that are then translated into corresponding motor actions in downstream midbrain areas such as the periaqueductal gray (PAG). Hypothalamic neurons also communicate with midbrain and hindbrain areas associated with reinforcement (such as the ventral tegmental area (VTA)) and arousal (including the locus coeruleus (LC) and dorsal raphe (DR)), and receive contextual and experience-dependent modulation from cortical circuits^4,5,38,47,164,198^. Cocaine- and amphetamine-regulated transcript (*Cart*)-positive neurons in the Edinger-Westphal nucleus (EWN) modulate maternal nesting during pregnancy^164^. Molecularly defined neuronal populations within in each region with established roles in rodent parental behaviour are shown (molecular markers for these populations are shown in the circles). The arrows represent experimentally supported anatomical or functional relationships inferred from tracing, activity mapping and circuit perturbation studies^12,14,16,17,32,35,37,39,40,46,47,51,53,59,64–66,70,82,83,87,89–91,105,111– 113,148,164,188,190,198,200,217,239^. Solid arrows indicate well-supported connections, whereas dashed arrows denote pathways that have been proposed (but lack experimental support) or are incompletely resolved. 5HT, serotonin; A1, primary auditory cortex; AHA, anterior hypothalamic area; AHi, amygdalohippocampal area; *Agrp*, agouti-related peptide; AOB, accessory olfactory bulb; ARC, arcuate nucleus; *Avp*, arginine vasopressin; AVPV, anteroventral periventricular nucleus; BLA, basolateral amygdala; BNST, bed nucleus of the stria terminalis; *Brs3*, bombesin receptor subtype 3; *Calcr*, calcitonin receptor; *Camk2*, calcium-calmodulin kinase II; *Calb1*, calbindin; CeA, central amygdala; *Crh*, corticotropin-releasing hormone; *Esr1*, oestrogen receptor alpha; *Gal*, galanin; IC, inferior colliculus; LC, locus coeruleus; LHA, lateral hypothalamic area; LHb, lateral habenula; LS, lateral septum; MeA, medial amygdala; MOE, main olfactory epithelium; mPFC, medial prefrontal cortex; NAc, nucleus accumbens; NAd, noradrenaline; *Npy*, neuropeptide Y; OB, olfactory bulb; OFC, orbitofrontal cortex; *Oxt*, oxytocin; PA, posterior amygdala; PeFA, Perifornical area; PIL, posterior intralaminar thalamus; *Prlr*, prolactin receptor; PVN, paraventricular nucleus of the hypothalamus; RetF, reticular formation; RRF, retrorubral field; S1, primary somatosensory cortex; SC, superior colliculus; TeA, temporal association area; *Th*, tyrosine hydroxylase; *Tip39*, tuberoinfundibular peptide of 39 residues; *Trpc5*, transient receptor potential cation channel 5; *Ucn3*, urocortin 3; *Vgat*, vesicular GABA transporter; *Vglut*, vesicular glutamate transporter; VMH, ventromedial hypothalamus; VNO, vomeronasal organ; *Npy2r*, neuropeptide Y receptor 2.

Rather than being organised around a single “parenting centre”, modern circuit approaches support a **distributed architecture** (Fig. 1). Pro-parental circuits promote approach towards young and the execution of caregiving sequences, whereas agonistic (anti-parental) circuits suppress care or drive avoidance and attack, depending on internal state and sensory context. Cell-type- and pathway-specific manipulations, together with in vivo recordings, are increasingly revealing how this network evaluates infant cues, organises caregiving actions, and suppresses mutually incompatible behaviours.

### Evaluating infant cues

A prerequisite for parenting is that infant-derived sensory cues are detected and assigned behavioural meaning. These cues are multisensory and include chemosensory, auditory, tactile and visual signals (Fig. 1)^5^. Importantly, deprivation of a single sensory modality has little effect on maternal responses, whereas disruption of two or more senses produces strong deficits, suggesting that pup recognition depends on the integration of multiple, partially redundant infant cues^29^. In rodents, however, these modalities do not contribute equally and are sequentially engaged as the adult approaches offspring. Auditory and volatile olfactory cues are important for detecting and localising pups at a distance, whereas contact chemosensory and tactile cues become relevant during close-range investigation and direct physical interaction^30–33^. Because most laboratory studies of parental behaviour in rodents are performed in darkness or low light, the contribution of vision is less well understood.

Auditory cues provide an important route by which pup distress calls are detected and translated into orienting and retrieval behaviours. In mice, pup ultrasonic vocalisations (USVs) are represented in primary auditory cortex (A1), where their encoding is strongly shaped by parental state and experience^34,35^. In females, only the left A1, together with the temporal association cortex (TeA), is required for the recognition of pup vocalisations and for efficient auditory-guided retrieval^34,36,37^. These auditory signals affect the hypothalamic circuitry that coordinates maternal behaviour via the posterior intralaminar thalamus (PIL), which relays information about pup calls to hypothalamic oxytocin neurons^38^. A recent preprint has reported that a parallel A1–inferior colliculus (IC) pathway also contributes to USV-driven pup retrieval in female mice^39^.

Chemosensation provides a second major route for infant detection and evaluation. Pup odours are detected by the main olfactory epithelium (MOE) and processed through the main olfactory bulb (OB). These cues contribute to pup localisation and approach behaviour, and disruption of MOE or OB function impairs maternal behaviour, in particular retrieval^40–43^. From the OB, olfactory information is relayed to cortical and amygdalar targets, including the medial amygdala (MeA) and bed nucleus of the stria terminalis (BNST), which connect chemosensory pup representations to hypothalamic parental circuits^44–48^. In sheep, bonding between ewe and lamb depends on intact olfactory processing: transient lidocaine inactivation of the MeA during the first hours after birth disrupts subsequent lamb-odour recognition, demonstrating that olfactory input to the MeA contributes to offspring-specific memory formation^49,50^. In female mice, olfactory pup cues can also shape auditory pup representations in A1 through experience-dependent recruitment of basolateral amygdala (BLA) inputs carrying olfactory information from upstream olfactory cortical and amygdalar regions^51^. These findings suggest that olfactory and auditory pup cues are integrated in auditory cortex to support localisation of pups before direct contact.

At closer range, non-volatile contact chemosensory cues detected by the **vomeronasal organ** (VNO) become particularly relevant (Fig. 1)^30–33^. In sexually naïve male mice, surgical or genetic ablation of the VNO abolishes pup-directed aggression and unmasks caregiving^52,53^, showing that VNO input normally promotes infant-directed aggression. These cues — which include pup salivary chemosignals^32^ — are relayed via the accessory olfactory bulb (AOB) to the MeA, where information from the main olfactory and vomeronasal pathways can thus be integrated^54,55^. In male rats, MeA lesions unmask parental care in otherwise infanticidal animals^56^. It is possible that pup-directed aggression involves MeA outputs to hypothalamic aggression centres, including the anterior hypothalamic area (AHA) and ventromedial hypothalamus (VMH) (areas known to be involved in aggressive behaviours^57,58^); however, direct evidence implicating these pathways specifically in pup-directed aggression is currently lacking. Nevertheless, the MeA is not simply an ‘anti-parental’ node: in males, it contains molecularly distinct populations that are associated with either pup care or male-male aggression^59^. Optogenetic activation of posterodorsal MeA GABAergic neurons promotes pup grooming in female mice, whereas in males, low-intensity stimulation of these neurons similarly promotes pup grooming but stronger activation can trigger infanticide^46^. Thus, chemosensory cues are likely to bias behaviour in opposite directions depending on both the identity of the MeA neuronal populations recruited and the level of their activation.

Finally, tactile cues become relevant once the adult has made direct physical contact with the infant^32^. Compared with auditory and chemosensory pathways, much less is known about which tactile characteristics promote caregiving (Fig. 1). In female rats, perioral anaesthesia impairs pup retrieval, and it is likely that the tactile information driving this behaviour is mediated via the infraorbital nerve^60,61^. In male mice, tactile detection of physical pup features (such as limbs) contributes to pup-directed aggression, together with contact chemosensory cues^32^. It has recently been shown that shown that mechanosensory inputs are integrated with hypothalamic state signals in downstream areas to gate the execution of mating behaviours ^62^; thus it is possible that a similar integration may drive parental motor actions. However, at present, the best-resolved tactile circuits are those involved in suckling and nursing. In rats, calbindin- and TIP39-expressing neurons in the PIL are activated by suckling and contribute to oxytocin and prolactin release, likely through projections to the paraventricular nucleus of the hypothalamus (PVN) and arcuate nucleus (ARC)^63–65^. In addition, tyrosine hydroxylase-expressing neurons in the anteroventral periventricular nucleus (AVPV) project directly to oxytocin neurons in the PVN and are recruited during maternal interactions, providing a complementary hypothalamic pathway through which infant stimuli can promote oxytocin release and caregiving^66^. PIL TIP39-expressing neurons also innervate neurons in the medial preoptic area (MPOA) of the hypothalamus that express galanin (*Gal*) (MPOA^*Gal*^ neurons) of the hypothalamus — a key hub within parental circuitry^47,53^ — providing a pathway through which pup contact can influence central parenting circuits (Fig. 1)^64,67^. At the cortical level, prolonged nursing induces plasticity in primary somatosensory cortex (S1)^68^, consistent with the idea that repeated tactile interactions refine pup representation and stabilise caregiving responses over time.

How pup-derived sensory signals are represented once they reach the central parenting circuits described in the next section is poorly understood. In mother mice, concurrent pup odour and USVs, which synergistically promote approach, induce greater *Fos* activation in both the BNST and MPOA than either cue alone^69^. More recent projection-specific studies reveal partially dissociable responses to different sensory cues in female mice: pup distress vocalisations activate lateral habenula (LHb) neurons receiving BNST input, and these neurons encode associated negative affect and are activated during pup retrieval^70^, whereas projections from MPOA^*Gal*^ neurons to the hypothalamic perifornical area (PeFA) respond to live pups and pup odour, but not to pup-call playback or pup-like objects^71^. Thus, distinct circuit elements within the BNST and MPOA may encode different infant features.

### Pro- and anti-parental central systems

Once infants have been detected, the brain must assign them behavioural meaning: should caregiving be promoted, or should it be suppressed and **agonistic behaviour** executed instead? A first stage of this evaluation process appears to occur within (extended) amygdala circuits. The BNST — which is strongly interconnected with the MeA, hypothalamus and olfactory regions — seems to be particularly important in converting pup-related sensory signals into motivational states. In postpartum female rats, lesions or pharmacological inhibition of the BNST disrupt pup retrieval and pup-directed care^72,73^, while reversible BNST inactivation impairs both the onset and maintenance of maternal behaviour in sheep^74^. Further downstream, the LHb has emerged as an important node in this circuit: pup distress vocalisations recruit BNST–LHb projections^70^, and lesioning or chronically silencing the LHb in primiparous dams disrupts pup retrieval and nest building^75^.

At the centre of the pro-parental network lies the MPOA, which acts as a hub for the initiation and organisation of caregiving (Fig. 1). Classical lesion studies showed that damage to the MPOA abolishes retrieval, grooming and nest building in female rats without producing gross locomotor or perceptual deficits^76,77^ — findings that have since been replicated in mice^78,79^ marmosets^80^ and sheep^74^. Conversely, electrical activation of the MPOA or local delivery of hormones such as oestradiol and prolactin can elicit maternal-like responses in virgin females and males^4,81^. More recent work has refined this classical view by revealing molecularly and projection-defined MPOA subpopulations that control discrete aspects of caregiving^14,17,18,46,47,53,70,79,82,83^.

Among these, MPOA^GAL^ neurons are strongly engaged during parenting; selective ablation of these neurons reduces pup-directed care, whereas activation suppresses aggression in male mice and promotes pup grooming in both sexes^47,53^. Oestrogen receptor-α (*Esr1*)-expressing MPOA neurons (MPOA^*Esr1*^ neurons), which make up approximately 80% of MPOA^*Gal*^ neurons^84^, are particularly implicated in pup approach and retrieval, and their activity rises before action initiation^82,85^. However, this population contributes to several behaviours and physiological functions, including parenting, mating and torpor^82,85,86^. Another population, calcitonin receptor-expressing MPOA neurons (MPOA^*Calcr*^ neurons), are recruited during both maternal and alloparental interactions^87^. In mothers, 70–85% of MPOA^*Calcr*^ neurons are *Esr1*-positive whereas a smaller proportion (~20%) are *Gal*-positive^87^. Chemogenetic activation of MPOA^*Calcr*^ neurons reduces infanticide and promotes pup-directed care, whereas silencing disrupts maternal behaviour^87^. The MPOA thus comprises multiple partially overlapping, molecularly defined neuronal populations that promote caregiving. The primary function of MPOA^*Gal*^ neurons — like that of other hypothalamic populations such as *Esr1*-expressing neurons in the ventrolateral part of the VMH (VMHvl^*Esr1*^ neurons) — may be less to encode specific actions than to represent an affective instinctive state that biases behavioural selection toward context-appropriate actions^88^.

The pro-parental neuronal populations are opposed by dedicated systems that suppress care and instead promote avoidance or pup-directed aggression. Urocortin-3-expressing neurons in the PeFA (PeFA^*Ucn3*^ neurons) promote infant neglect and mild aggression in males and females, and receive monosynaptic inputs from stress- and parenting-associated populations — including corticotropin-releasing hormone (*Crh*)-expressing neurons in the PVN (PVN^*Crh*^ neurons) and MPOA^*Gal*^ neurons^89^. Different PeFA^*Ucn3*^ neuron projections seem to control distinct aspects of agonistic behaviour: whereas PeFA–VMH projections suppress pup investigation, PeFA–lateral septum (LS) stimulation promotes repulsion, and PeFA–amygdalohippocampal area (AHi) stimulation promotes pup-directed aggression^89^. Consistent with this, excitatory AHi–MPOA projections drive infant-directed aggression and suppress parenting in male mice^90^. Similarly, neurons in the rhomboid nucleus of the BNST (BNSTrh) have been found to be recruited during infanticide, and lesions of this structure reduce infanticidal behaviour in virgin males^79^. More recent work identified a reciprocal BNST–MPOA circuit that acts as a switch between caregiving and aggression. In both male and female mice, *Esr1*-expressing neurons in the principal BNST (BNSTpr^*Esr1*^ neurons) promote infant-directed aggression, whereas MPOA^*Esr1*^ neurons promote caregiving (Fig. 1)^14,91^. These two populations are coupled by mutual inhibition such that activation of one suppresses the other. Reproductive state biases behavioural output — caregiving versus aggression — by shifting the balance of activity within this antagonistic circuit (**Box 2**)^14,91^. Where and how pro-parental and agonistic circuits communicate elsewhere in the brain remains to be shown.

### Reinforcement and action

Caregiving often entails substantial energetic and opportunity costs, and competes with immediate needs such as feeding, rest and self-preservation, as discussed below. For parental behaviour to dominate action selection, infants must become strongly rewarding and infant-directed interactions must acquire strong reinforcing value. Classical operant studies in female rats showed that animals will lever-press to gain access to pups, indicating that infants themselves are powerful reinforcers^92^. Remarkably, the reinforcing value of pups, measured by lever pressing frequency, can exceed that observed for cocaine in the same paradigm, underscoring the exceptional hedonic valence of offspring cues^93^. However, not all infant cues are uniformly attractive — pup distress vocalisations can carry aversive valence in virgin females for instance^70^. How infant cues acquire positive motivational value remains an important open question. A longstanding model proposes that the MPOA acts as a parental motivational hub that enables pup stimuli to engage the mesolimbic dopamine (DA) system^5^. In this model, MPOA activity recruits ventral tegmental area (VTA) projections to the nucleus accumbens (NAc), thereby endowing infant stimuli with incentive salience and promoting pup-directed approach and caregiving.

Consistent with this framework, dopaminergic pathways centred on the VTA and NAc are central to reinforcement and performance during parenting (Fig. 1)^94,95^. In postpartum rats, DA depletion reduces the responsiveness of female rats to pups and disrupts goal-directed maternal behaviours such as retrieval^96,97^. Similar deficits occur following transient VTA inactivation^98^. More recent work in pup-exposed virgin female mice suggests that DA signals, as in many other contexts, have reinforcement-like properties during pup interactions: the activity of VTA dopaminergic neurons (VTA^DA^ neurons) peaks during pup contact and then decreases with experience as retrieval becomes more efficient, and temporally precise VTA^DA^ neuron inhibition during pup contact decreases future retrieval performance^83^.

Further downstream, the NAc shell appears particularly important for maternal performance. Lesions of this structure or pharmacological blockade of DA receptors therein impair pup retrieval and grooming in postpartum female rats^99,100^. Moreover, individual differences in maternal behaviour correlate with stronger DA signals during mother-pup interactions^101^, consistent with a role for accumbal DA tone in sustaining maternal engagement^102^. Human imaging studies similarly show that infant cues recruit ventral striatal and VTA circuitry in mothers, often more strongly in response to one’s own infant than to unfamiliar infants, and that these responses correlate with measures of parent-infant attachment^103,104^. Recent work in mice further indicates that cortical inputs, including orbitofrontal projections, shape VTA^DA^ neuron dynamics during caregiving^105^. Together, these findings suggest that mesolimbic circuits reinforce successful infant-directed interactions, whereas hypothalamic populations such as MPOA^*Gal*^ neurons may encode the underlying parental affective or motivational state.

This parental state must then be translated into specific motor sequences. The periaqueductal gray (PAG) is a major downstream midbrain structure that contributes to the motor and autonomic implementation of caregiving as well as other instinctive responses^47,106^. Lesion studies in lactating rats support a modular organisation: caudolateral and ventrolateral PAG lesions reduce suckling-induced kyphosis (a characteristic, arched-back nursing posture) and alter maternal defence (aggression towards intruders threatening the litter), whereas more rostral lesions disrupt pup retrieval^107–110^. At the circuit level, projections from MPOA^*Gal*^ neurons and vesicular GABA transporter (*Vgat*)-expressing MPOA neurons (MPOA^*Vgat*^ neurons) to the PAG drive pup grooming and retrieval, respectively, in both sexes^47,111^. In contrast, a more posterior substantia innominata (SI)–PAG pathway promotes pup-directed aggression in males^112^. MPOA outputs may also recruit brainstem motor nuclei in the reticular formation, potentially via the retrorubral field^113^, but these pathways remain less well characterised.

### Cortical contributions

Cortical circuits provide contextual and experience-dependent control over the largely subcortical parental network. Sensory cortex is particularly important for the representation of infant cues: as discussed above, pup vocalisations are encoded in A1 in a strongly state- and experience-dependent manner^34,114^, allowing prior parental experience and oxytocin signaling to shape auditory-guided retrieval. Frontal cortical regions contribute to the flexible organisation of parental behaviour under changing environmental demands. In rats, disruption of the medial prefrontal cortex (mPFC) impairs the sequencing of maternal actions, including retrieval and nesting behaviours^115,116^. In mice, mPFC neurons that project to the MPOA are preferentially engaged during maternal defence rather than caregiving, and perturbing this pathway disrupts threat-related responses^117^. Similarly, excitatory orbitofrontal cortex (OFC) projections promote experience-dependent alloparenting in virgin females, likely through modulation of VTA^DA^ neurons^105^. Human imaging studies likewise implicate medial prefrontal and cingulate networks in responses to infant cues and distress^118,119^. Together, these findings suggest that cortical regions refine sensory representations of offspring and enable parental behaviour to be flexibly adjusted to experience and immediate environmental context.

## Parental behaviour across the lifespan

**Fig. 2:**
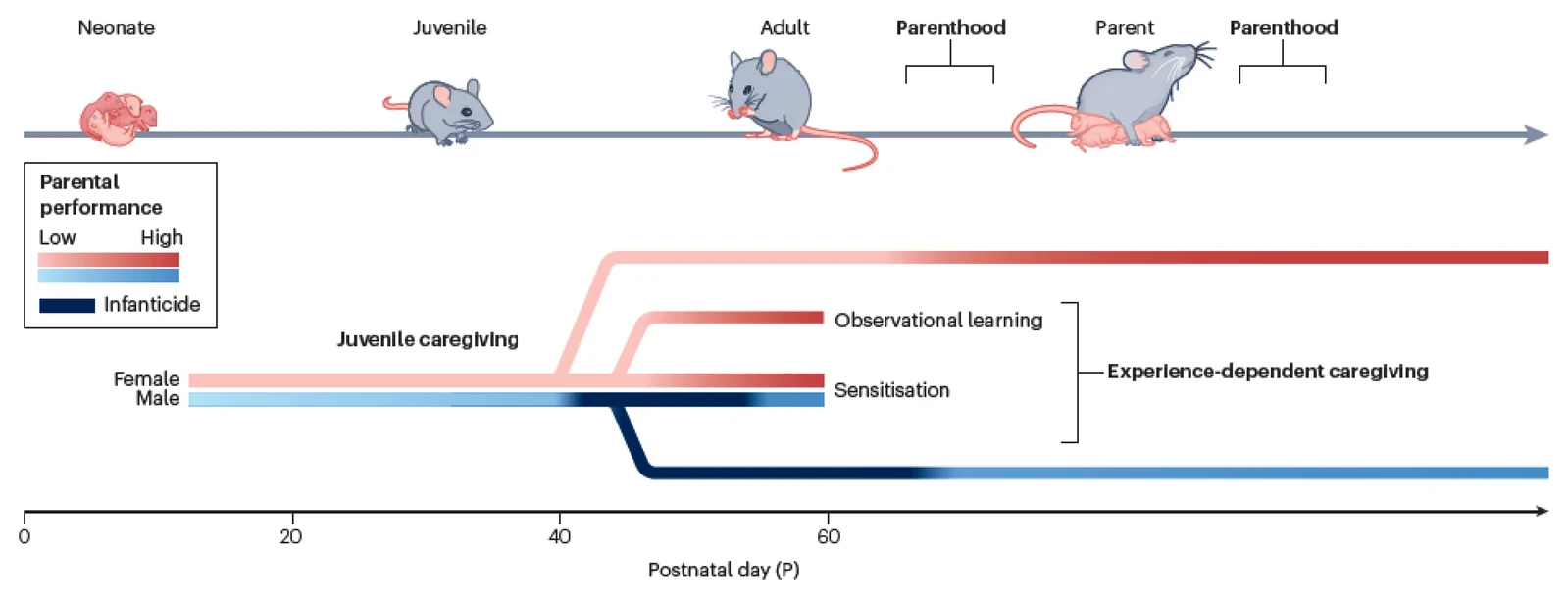
Parental behaviour across the lifespan. Schematic overview of life stage–dependent routes through which parental and alloparental behaviour can emerge in mice, including juvenile caregiving, experience-dependent behaviour, and parenthood. In juveniles, caregiving emerges before sexual maturity in both sexes as a developmentally regulated form of alloparental behaviour^19,128,129^. In adulthood, parental behaviour can be acquired before parenthood through experience-dependent mechanisms, including repeated pup exposure (sensitisation) and observational learning from experienced caregivers^16,122,139^. Parenthood is the major route into high parental performance, with pregnancy, parturition and lactation promoting maternal care, and mating, cohabitation and/or prior reproductive experience facilitating paternal behaviour in naturally biparental species and contexts in which males contribute to care^4,17,52,81^. The red and blue trajectories indicate relative parental performance — i.e., the likelihood that parental behaviour is expressed and the robustness with which it is performed — in females and males, respectively, across postnatal life. Dark blue shading indicates periods during which pup-directed aggression or infanticide can occur in adult males before the transition to paternal behaviour^240–243^. Approximate timing is shown for laboratory mice; this may vary in other species and/ or conditions.

In addition to the cortical modulation of parental behaviour, substantial behavioural flexibility is already embedded within subcortical parenting circuits. Similar caregiving behaviours can be expressed by juveniles, sexually inexperienced (virgin) adults, mothers, fathers and post-reproductive individuals, indicating that functional parenting circuits are present throughout life but recruited according to behavioural need^5,11,15,19,120,121^. Developmental stage, endocrine state and experience all act on a shared circuit scaffold to shift the balance between pro-parental and anti-parental pathways^11,14,19,47,91^. A lifespan perspective (Fig. 2) provides a useful organising principle for understanding this parental circuit plasticity. During juvenile development in mice, rapid circuit maturation initiates caregiving towards younger siblings^19^; in sexually naïve adults, repeated pup exposure and social observation can promote the function of the same circuit through experience-dependent plasticity^15,35,122^. Pregnancy and parturition are accompanied by profound endocrine changes that drive large-scale brain remodelling and reorganise these circuits into a stable caregiving state^11,123–125^. Finally, investigations into repeated parenthood and ageing may reveal whether parental plasticity accumulates across successive reproductive experiences, and how long-lasting these changes are.

### Onset of alloparental behaviour in juveniles

**Fig. 3:**
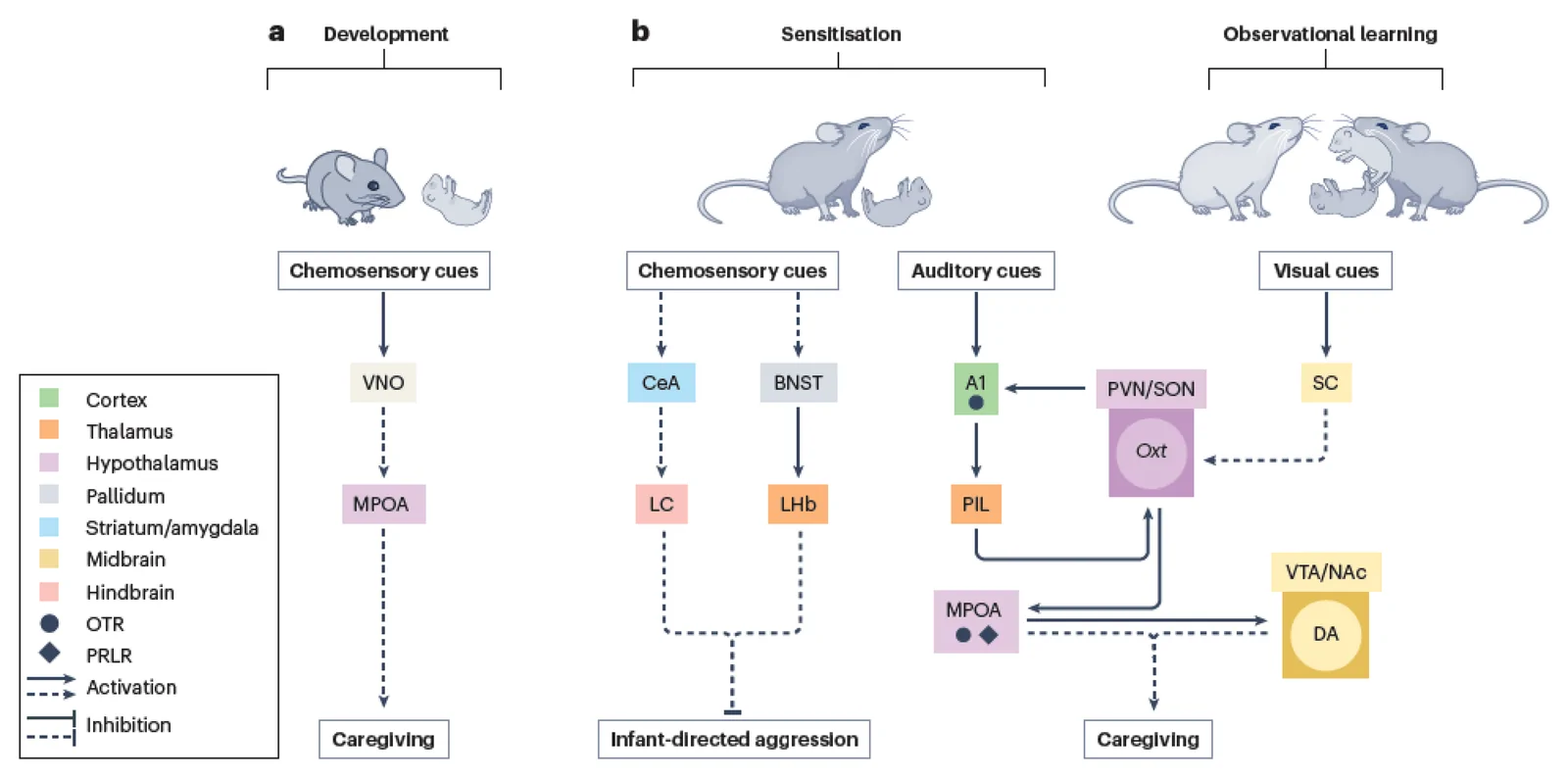
Caregiving outside parenthood. Caregiving can emerge outside parenthood through developmental, experience-dependent and socially transmitted mechanisms. **a**, In juvenile mice, alloparental behaviour appears during the first month of postnatal development. Pup-derived chemosensory cues are routed through vomeronasal pathways to the medial preoptic area (MPOA), where developmental circuit maturation enables caregiving behaviour^132^. A recent preprint suggests that this transition depends on microglia-mediated remodelling of synaptic inputs onto parental MPOA neurons^19^. Dashed arrows indicate incompletely resolved connections or functional relationships. **b**, In adult virgin mice, caregiving can be acquired through repeated pup exposure (sensitisation) or observation of experienced caregivers. Sensitisation promotes caregiving, in part, through chemosensory circuits that suppress infant-directed aggression^70,145^. In addition, pup vocalisations recruit oxytocin (*Oxt*) neurons in the paraventricular nucleus (PVN), and potentially the supraoptic nucleus (SON), leading to oxytocin release that promotes plasticity in sensory and motivational circuits and supports subsequent caregiving responses^16,34,38^. Similarly, observation of maternal behaviour recruits oxytocinergic pathways that facilitate subsequent pup retrieval and care^148^. Within the MPOA, oxytocin receptors (OTR) and prolactin receptors (PRLR) may provide additional routes through which neuroendocrine signals facilitate caregiving: repeated pup exposure recruits OTR-expressing neurons, whereas pup-experienced virgin females show enhanced PRLR signalling^144,147^. Molecularly defined neuronal populations with established roles in parental behaviour are shown (molecular markers shown in circles). Arrows represent experimentally supported anatomical or functional relationships inferred from tracing, activity mapping and circuit perturbation studies^16,19,34,38,47,70,83,132,138,144–146,148^. Solid lines indicate well-supported connections, whereas dashed lines denote proposed or incompletely resolved pathways. A1, primary auditory cortex; BNST, bed nucleus of the stria terminalis; CeA, central amygdala; DA, dopamine; LC, locus coeruleus; LHb,lateral habenula; PIL, posterior intralaminar thalamus; NAc, nucleus accumbens; *Oxt*, oxytocin; SC, superior colliculus; VTA, ventral tegmental area; VNO, vomeronasal organ.

In cooperative breeders, including humans, some non-human primates, carnivores and rodents, juveniles commonly interact with, and help care for infants, indicating that functional parental circuits predate adulthood^126,127^. Juvenile alloparenting behaviour has also been observed in rats^128,129^, prairie voles^130,131^ and mice^120,121^. A recent preprint has shown that, in laboratory mice, alloparental behaviour emerges abruptly between postnatal day 14 and 15, independently of prior social experience or ovarian hormones^19^. It was reported that this transition coincides with the onset of pup-responsive activity in MPOA^*Gal*^ neurons and is driven by microglia-mediated remodelling of their synaptic inputs^19^, which prunes an immature network into an adult-like configuration permissive for caregiving. In parallel, the maturation of the preoptic area is shaped by vomeronasal sensory inputs (Fig. 3a)^132^. Juvenile caregiving thus abruptly emerges through experience-independent remodelling of sensory inputs into parenting-controlling hypothalamic neurons.

Whereas both male and female juvenile mice are alloparental, infant-directed behaviours diverge in virgin adults: females often show spontaneous parental behaviour, whereas males typically exhibit pup-directed aggression^5^. This suggests that puberty reshapes parental circuits in a sex-specific manner. Indeed, it has been shown that prepubertal **gonadectomy** impairs subsequent parental performance in both male and female adults^133^, and treatment of males with oestradiol or dihydrotestosterone (DHT) promotes later pup-directed aggression and alters synaptic transmission in the BNSTrh, consistent with a potentiation of anti-parental pathways^134^.

### Learning to care: experience-driven parenting

Repeated pup exposure can transform an initially indifferent, avoidant or even aggressive adult into a reliable caregiver, a process called **sensitisation**. This has been most clearly demonstrated in virgin female rodents, in which repeated pup exposure induces retrieval, licking and nursing^15,122,135,136^. Sensitisation also occurs in males^137,138^. In addition, caregiving can be acquired through **observational learning**, as naïve females exposed to maternal behaviour show increased responsiveness to pup cues and parental performance^34,35^. Social experience can thus recruit latent caregiving circuits (Fig. 3b).

Classical studies showed that sensitisation can induce caregiving in **nulliparous**, ovariectomised or **hypophysectomised** females, indicating that this process does not require ovarian or pituitary hormones^122,135,136,139^. Pup odours and vocalisations seem to contribute to this process^33,140,141^, and — once established — these changes can persist for weeks to months, consistent with a form of maternal memory^142,143^. Sensitisation seems to engage the core parental network rather than a distinct system (Fig. 3b)^15^. In female mice, repeated pup exposure increases activity in the MPOA^144^ and is accompanied by enhanced oxytocin signalling: oxytocin neuron activity and peptide levels rise in the PVN and supraoptic nucleus (SON), and sensitisation is associated with increased activation of oxytocin receptor-expressing neurons in the MPOA^144^. In line with this, artificial activation of oxytocin expressing PVN neurons (PVN^*Oxt*^ neurons) is sufficient to promote pup retrieval in virgin females^144^. Furthermore, a recent preprint has reported that oxytocin enhances pup-call responses in A1^39^. In parallel, sensitisation weakens aversion-related pathways, including central amygdala (CeA)–locus coeruleus (LC) projections^145^, and BNST–LHb projections that encode negative pup valence in naïve mice^70^. It also recruits reward-related circuits, engaging reinforcement-signalling VTA^DA^ neurons and, in males, increasing activity in the NAc, consistent with a shift in the motivational value of pups^83,138^. In females, prolactin may further enhance parental responsiveness and sensitivity to pup cues^146,147^.

Observational learning appears to rely on mechanisms that partially overlap with those driving sensitisation (Fig. 3b). Co-housing virgin females with dams accelerates pup retrieval, and this learning depends in part on visual observation as well as shepherding — the active guidance of a naïve individual towards pups or the nest by the dam^16^. Oxytocin signalling plays a central role in this process: PVN^*Oxt*^ neurons are engaged during observational learning, and both SC–PVN and PVN–A1 projections facilitate the social acquisition of parental behaviour^16,34^. Strikingly, a recent preprint has shown that even viewing videos of adult conspecifics in a non-parental context is sufficient to promote parental behaviour via mechanisms that involve SC–PVN^*Oxt*^ projections, suggesting that social arousal itself can recruit latent caregiving circuits independently of direct pup experience^148^. Comparable forms of observational learning have not been extensively studied in male mice and other rodent species. However, given the importance of social interactions in many biparental and cooperatively breeding mammals, similar mechanisms may contribute to the acquisition of caregiving beyond laboratory mice.

### Reproductive experience and parenthood

**Fig. 4:**
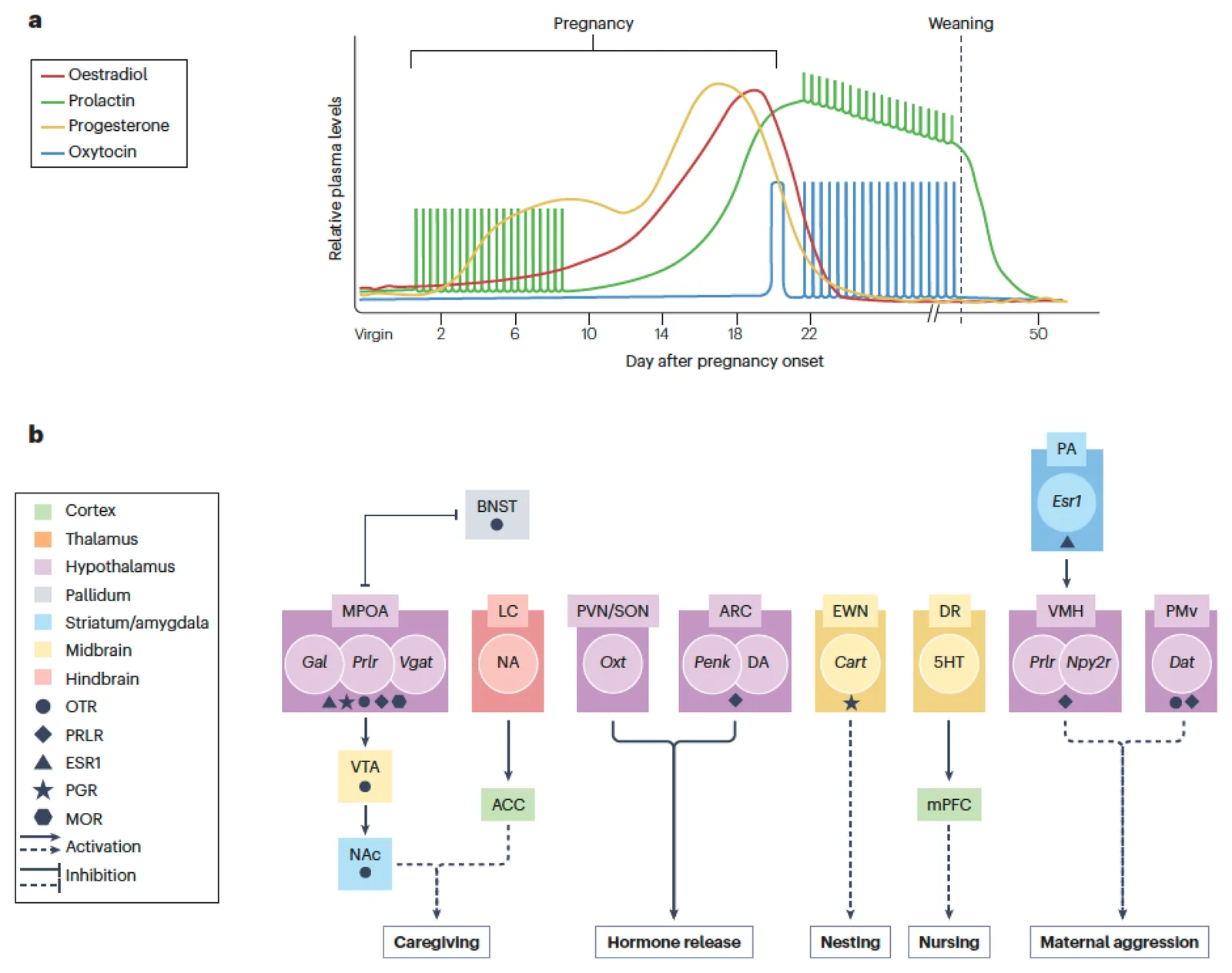
Hormone action in parenting circuits during motherhood. **a**, Schematic summary of relative blood plasma hormone levels across pregnancy, parturition, lactation and weaning. Oestradiol, progesterone, prolactin and oxytocin change dynamically across this period, with levels of ovarian steroid hormones rising during pregnancy and rapidly decreasing at parturition, followed by pulsatile, suckling-associated prolactin and oxytocin release during lactation^81,165^. Approximate timing shown for laboratory mice. **b**, These hormones act across multiple nodes of the parenting network, including the medial preoptic area (MPOA), bed nucleus of the stria terminalis (BNST), ventral tegmental area (VTA) and nucleus accumbens (NAc), to promote maternal caregiving behaviour^4,5,11,47,82,179,203^. During lactation prolactin drives a neurotransmitter phenotype switch in arcuate (ARC) tuberoinfundibular neurons from dopamine (DA) to met-enkephalin (*Penk*) to support sustained prolactin release^205^. Other hormone-sensitive or neuromodulatory pathways that shape specific components of maternal behaviour include midbrain pathways implicated in nesting^164^ and nursing^200^ and hypothalamic circuits that contribute to maternal aggression and defensive behaviour^187–189^. Brain regions, genetically defined neuronal populations and arrows indicate experimentally supported anatomical or functional relationships inferred from tracing, activity mapping and perturbation studies^83,151,164,169,184,187–190,197–202,205^. Solid lines indicate experimentally supported anatomical or functional relationships; dashed lines indicate proposed or incompletely resolved connections. 5HT, serotonin; *Cart*, cocaine- and amphetamine-regulated transcript; *Dat*, dopamine transporter; ESR1, oestrogen receptor alpha; EWN, Edinger-Westphal nucleus; *Gal*, galanin; MOR, mu opioid receptor; OTR, oxytocin receptor; PA, posterior amygdala; PGR, progesterone receptor; PMv, ventral premammillary nucleus; PRLR, prolactin receptor; *Vgat*, vesicular GABA transporter; VMH, ventromedial hypothalamus; *Npy2r*, Neuropeptide Y receptor 2.

Experience alone can thus recruit high levels of parental behaviour. In motherhood, the same circuitry is engaged through large-scale endocrine remodelling, stabilising caregiving as a dominant behavioural state (Fig. 4). Most mechanistic insights into the underlying neural processes come from studies in females, whereas the neural basis of paternal behaviour and fatherhood — particularly in naturally biparental species — remain far less well understood (**Box 2**).

#### Pregnancy

The transition into parenthood begins at conception. Across mammals, hormonal fluctuations ― particularly of oestradiol, progesterone, prolactin, and oxytocin ― shape physiology and behaviour during pregnancy and the postpartum period (Fig. 4). The receptors for these hormones are enriched in key nodes of parenting circuits^124^. During motherhood, the brain also undergoes widespread grey matter volume changes which track hormone levels and correlate with subsequent responsiveness to infant cues and quality of mother–infant attachment (reviewed in ^123,124^).

In mice, prolactin secretion increases during early pregnancy before placental lactogens become the predominant ligands for the prolactin receptor (PRLR)^149,150^. Activation of hypothalamic PRLR signalling promotes infant approach and caregiving during pregnancy and has also been implicated in caregiving in males^151–153^. Together, these findings indicate that lactogenic hormones act as early organisers of the parental state.

As pregnancy progresses, progesterone and oestradiol levels rise sharply (Fig. 4a), and robust parental behaviour emerges in female mice, reaching levels by late gestation that are indistinguishable from those of mothers^11^. In rats, this transition is accompanied by increased progesterone receptor (PGR) expression in the MPOA^154^. Classical endocrine manipulations demonstrate that these hormonal dynamics are causally linked to the onset of maternal behaviour. In ovariectomised rats, oestradiol (but not progesterone) treatment shortens the latency to pup retrieval and care, whereas prior exposure to progesterone for 4–10 days sensitises females to lower doses of oestradiol, resulting in a more robust behavioural response^155^. Similarly, in mice, maternal behaviour is rapidly induced by oestradiol within hours of progesterone withdrawal, whereas prolonged oestradiol exposure following progesterone withdrawal impairs pup retrieval and nest building^156^. Together, these findings indicate that the transition to maternal behaviour depends not simply on the absolute concentrations of progesterone and oestradiol, but on their temporal sequence and relative dynamics. One proposed mechanism is that sustained progesterone signalling primes MPOA neurons through PGR-dependent transcriptional and chromatin remodelling, thereby enhancing subsequent oestradiol-responsive gene expression^157^, although the precise molecular mechanisms remain incompletely understood. Consistent with this model, coordinated late-gestation trajectories of progesterone and oestradiol predict subsequent maternal sensitivity in humans^158^.

Recent circuit-level work has begun to explore how these hormones act on parental circuitry. Disrupting oestradiol signalling in mice impairs maternal caregiving: global or MPOA-specific ablation of *Esr1* produces marked deficits in pup retrieval, nursing and grooming and can increase infanticide^82,159,160^, while natural variation in MPOA *Esr1* levels correlates with subsequent maternal performance^161–163^. Consistent with direct hormonal action on parenting-relevant neurons, MPOA^*Gal*^ neuron-specific deletion of either *Esr1* or *Pgr* abolishes the pregnancy-associated increase in parental behaviour and leads to persistent caregiving deficits^11^. Oestradiol and progesterone induce complementary forms of plasticity in MPOA^*Gal*^ neurons: oestradiol reduces baseline firing while increasing neuronal excitability, whereas progesterone promotes dendritic spine formation and recruits excitatory synaptic inputs^11^. This results in sparser MPOA^*Gal*^ neuron population activity in vivo, together with stronger, more selective responses to pup stimuli. Ovarian hormones thus remodel a central node in parental circuits during pregnancy, thereby preparing the system for caregiving. Consistent with this anticipatory role, progesterone also promotes nest building through cocaine and amphetamine regulated transcript (*Cart*)-expressing neurons in the Edinger–Westphal nucleus in the midbrain (Fig. 4b)^164^.

#### Parturition and parenthood

Parturition marks a rapid endocrine transition that consolidates the maternal state established during pregnancy. Placental expulsion causes a sharp decline in progesterone and oestradiol levels, whereas ongoing lactogenic signalling together with oxytocin helps stabilise caregiving after parturition^66,151,165^. Oxytocin release increases both peripherally, where it supports uterine contractions, and throughout the brain^166–168^. In parallel, oxytocin receptor (OTR) expression is transiently upregulated in regions such as the MPOA and BNST^169^, thereby increasing the sensitivity of parenting circuits to oxytocin. Pharmacological studies in rats and sheep show that central oxytocin administration facilitates maternal behaviour, whereas oxytocin blockade delays its onset^170–174^. Conversely, genetic loss of oxytocin or OTR in mice increases the latency to initiate nurturing, although once caregiving is established these deficits are surprisingly subtle^175–179^. Together, these findings indicate that oxytocin is particularly important for initiating maternal behaviour, whereas its role becomes less important once caregiving is established.

Classical studies in rats reported substantial maternal behaviour even after Caesarean delivery^180,181^, indicating that pregnancy itself preconfigures much of the maternal state. However, more recent work shows that Caesarean delivery delays pup retrieval and reduces pup grooming, effects that are partially rescued by exogenous oxytocin^182^, suggesting that parturition-associated signals further refine parental circuit function. This transition likely depends on **peripartum** astrocytic remodelling, as a recent preprint reported that retraction of astrocytic processes surrounding oxytocin neurons permits greater oxytocin release in response to offspring^183^.

Following birth, prolactin remains elevated to support milk production, while oxytocin is released in suckling-linked bursts to drive milk ejection^184^. Beyond these peripheral roles, prolactin directly regulates parental circuitry: global or MPOA-specific deletion of *Prlr* in mice abolishes maternal care while sparing lactation^151,185^.

Lactation is also accompanied by the emergence of maternal aggression, a protective component of the maternal behavioural repertoire in which mothers attack unfamiliar adult conspecifics to protect their offspring^186^. Maternal aggression is mediated by a distributed network that includes the posterior amygdala (PA), VMHvl and ventral premammillary nucleus (PMv)^187,188^. Recent work identified an excitatory projection from *Esr1*-expressing neurons in PA (PA^*Esr1*^) neurons to neuropeptide Y receptor 2-expressing neurons in VMHvl (VMHvl^*Npy2r*^) neurons as a key component of maternal aggression circuitry in mice^188^. During lactation, both PA–VMHvl synaptic transmission and VMHvl^*Npy2r*^ neuron excitability are enhanced, while oxytocin signalling increases the responsiveness of PA^*Esr1*^ neurons, collectively promoting robust maternal defence^188^. Prolactin exerts multiple, region-specific effects within these circuits. In the VMH, prolactin signalling restrains maternal aggression through *Prlr*-expressing neurons^189^. In parallel, prolactin and oxytocin transiently recruit a previously quiescent population of dopamine transporter-expressing PMv (PMv^*Dat*^) neurons, increasing their excitability and enabling the expression of maternal aggression at the expense of ongoing pup retrieval or nursing (Fig. 4b)^187^.

Oxytocin and vasopressin (AVP) provide additional neuromodulatory control across distributed sensory, hypothalamic, limbic and reward circuits to support maternal performance after birth. In mice, oxytocin signalling in the VTA enhances DA release in the NAc and correlates with individual differences in maternal grooming^190^. AVP exerts a parallel modulatory influence on parental behaviour: in rats, central AVP administration enhances caregiving, whereas blockade of its receptors — particularly V1a (AVPR1A) and V1b (AVPR1B) — reduces both maternal care and maternal aggression^191–195^. Consistent with this, genetic deletion of *Avpr1a* impairs maternal behaviour in Syrian hamsters (*Mesocricetus auratus*)^196^.

Monoamines and endogenous opioids provide further state-dependent modulation of parental circuits. In rats, DA release in the NAc correlates with maternal licking and pup grooming^101^, and blocking accumbal DA receptors disrupts caregiving^99^, in part via oxytocin-sensitive VTA–NAc projections^190^. Noradrenergic LC–anterior cingulate cortex (ACC) signalling supports maternal behaviour in mice^197,198^, while serotonin (5-HT) released from the rat dorsal raphe (DR) is required for nursing through actions on 5HT2A receptors in the mPFC rather than directly within the MPOA^199–201^. By contrast, endogenous opioid signalling appears to constrain caregiving: in rats, hypothalamic μ-opioid receptor expression decreases after parturition^202^, morphine suppresses maternal behaviour^203^, and opioid receptor antagonism enhances nursing^204^. Notably, tuberoinfundibular DA neurons in the ARC may switch to enkephalin release under high-prolactin conditions in mice, providing a potential mechanism by which endocrine state engages opioid modulation of parental circuits^205^. Several layers of hormonal and neuromodulatory control thus act together to promote the onset and stabilisation of robust caregiving behaviour during parenthood.

#### Long-term plasticity and parental experience

Reproductive experience can have enduring effects on parental behaviour and its underlying neural substrates. Prior pregnancy and caregiving accelerate the onset of maternal responses in subsequent reproductive cycles and can produce lasting changes in defensive components of care^206,207^. More broadly, pregnancy and motherhood induce persistent physiological and neural adaptations, although it remains unclear to what extent later maternal behaviour depends on retained circuit modifications versus endocrine changes occurring during each new pregnancy and parturition^123,208,209^.

Some of these adaptations persist well beyond the reproductive period itself. In rodents, parity is associated with lasting cellular and molecular brain changes^210^. In humans, childbirth is linked to persistent structural alterations, including a younger-looking brain-age profile in midlife^211^, and longitudinal imaging studies show sustained changes in grey matter volume, cortical thickness and white matter microstructure following pregnancy^212^. Together, these findings suggest that parenthood can leave lasting imprints on the brain.

In some species, these persistent changes may support continued caregiving roles beyond reproduction, although this has not been experimentally tested. In killer whales (*Orcinus orca*), post-reproductive females enhance grandoffspring survival and assume leadership roles during periods of resource scarcity^213,214^. Similarly, in Asian elephants (*Elephas maximus*), the presence of a grandmother improves calf survival and maternal reproductive success in daughters^215^. In humans, too, grandmaternal investment is associated with beneficial effects on descendant outcomes^216^, although these effects likely reflect a combination of neural, social and ecological mechanisms.

## Balancing care with competing drives

Parenting is costly, can increase exposure to danger, and — in some contexts — must be balanced with sexual behaviour to maximise lifetime reproductive success. Even in experienced parents, infant-directed behaviour is therefore continuously balanced against other urgent behavioural demands, including feeding, threat and stress avoidance, mating and rest. A central challenge is thus to understand how parental circuits communicate with other neural systems to organise this behavioural competition.

One of the clearest examples is feeding^12,217,218^. In mice, the presence of pups can suppress hunger-driven food intake^219^, and activation of excitatory MPOA neurons is sufficient to inhibit feeding^219^. This competition is bidirectional: food deprivation or activation of agouti-related peptide (AGRP) neurons in the ARC — which orchestrate the behavioural effects of hunger — can promote pup neglect or pup-directed or aggression in virgin female mice by inhibiting MPOA neurons through the release of GABA and neuropeptide Y (NPY)^12,217^. Importantly, both hormonal state and social experience shape this interaction. Fluctuations in progesterone-to-oestradiol hormone ratio across the oestrous cycle alter neuronal excitability in the MPOA, thereby altering the probability that hunger-evoked NPY release will suppress MPOA activity and promote pup-directed aggression^12^. In contrast, food deprivation does not induce pup-directed aggression in pup-sensitised virgin females^12,217^, indicating that prior social experience can stabilise caregiving in the face of metabolic challenge. Beyond the hypothalamus, fasting also increases NPY signalling through Y1 receptors in the mouse dorsal raphe nucleus, which contributes to the suppression of maternal care^220^. Together, these findings indicate that the balance between care and feeding is dynamically shaped by energetic need, endocrine state and prior social experience.

Caregiving must also be balanced against defensive responses to threat and stress. Stress can strongly disrupt parental behaviour, reducing pup retrieval, grooming and nursing while increasing avoidance, neglect and, in extreme cases, infant-directed aggression in female rats and mice^221–223^. Anti-parental neuronal populations such as PeFA^*Ucn3*^ neurons provide a route through which stress signals can suppress caregiving: these neurons receive input from both stress- and parenting-associated neuronal populations, including *Crh*-expressing neurons in the PVN and MPOA^*Gal*^ neurons, and their activation promotes infant neglect and aggression^89^. Direct manipulation of CRH signalling similarly suppresses parental behaviour. In ovariectomized, steroid-primed virgin female rats, intracerebroventricular CRH administration suppresses pup retrieval and grooming and, at higher doses, induces infanticide^224^. These effects are anatomically specific: CRH signalling in the MPOA impairs retrieval and especially nursing^225^, whereas in the anterior-dorsal BNST it induces maternal neglect^226^. Here, too, prior experience reduces both the magnitude and duration of this inhibition and prevents CRH-induced infanticide^224^. Motherhood provides an additional layer of resilience to stress. Parental behaviour in dams is remarkably resistant to perinatal stress and corticosterone treatment^227^, and defensive responses are reorganised to prioritize offspring protection: when exposed to threat, dams preferentially run toward an exposed pup and carry it to safety instead of fleeing, and this seems to be driven by mPFC projections to MPOA^*Esr1*^ neurons^117^.

A third dimension of behavioural competition is between parental care and mating. This is particularly evident during postpartum oestrus, when females can be simultaneously maternally and sexually motivated, and the relative expression of these behaviours depends on pup age, hormonal state and environmental context^5,228^. Although the VMHvl is considered the principal hypothalamic regulator of female sexual receptivity^229–232^, activation of MPOA neurons suppresses female sexual motivation after mating, suggesting that the MPOA may also contribute to coordinating parental and sexual motivational states^233^. Although little is currently known about local connectivity within the MPOA, local microcircuit organisation might contribute to switching between these competing behavioural states. Recent work also suggests that, in mice, there is a partial overlap between MPOA neurons associated with parenting and prosocial behaviour (helping behaviour directed at adult conspecifics)^234^, thus lending support to the longstanding hypothesis that prosocial behaviours build on neural systems originally evolved for parental care^235^. Similarly, in marmosets, partial overlap between parenting- and mating-associated MPOA neurons suggests that this organisation may reflect a conserved principle of shared hypothalamic control over social behaviour^80^.

More generally, parental circuits are shaped by numerous internal drives and motivational states, including hunger, stress, arousal, sexual motivation, sleep pressure and reproductive state. Where and how this high-dimensional state space is integrated in the brain and translated into context-appropriate social actions remains largely unresolved and represents one of the major outstanding challenges in understanding parenting and behaviour in general.

## Conclusions and perspectives

Recent work has substantially advanced our understanding of the neural basis of parental behaviour. Across life stages, a common, highly distributed circuit scaffold can be recruited in juveniles, non-parental adults, mothers and fathers, and may also support caregiving by post-reproductive individuals in some species. This circuit scaffold is profoundly shaped by endocrine state, social experience, developmental stage and competing motivational demands. Parental circuits are therefore both robust and flexible: robust in that they can generate caregiving with little or no prior experience, and flexible in that their output is continuously recalibrated according to internal state and external demands.

A central conceptual question is whether parental behaviour is controlled by a dedicated and molecularly distinct circuit, or whether it instead emerges from more general-purpose social-behavioural circuits shared with mating, aggression and prosociality. Recent evidence for overlap between parenting-related and other hypothalamic populations argues against a simple one-circuit-one-behaviour view^234^. In this framework, behavioural specificity may not arise from fully segregated circuit modules, but from how shared circuit elements are differentially recruited by sensory context and internal state. This question extends to what should count as part of a ‘parental circuit’: should inclusion require selective activity during parenting and necessity or sufficiency for caregiving, or is a modulatory role enough? This is particularly relevant for cortical and neuromodulatory systems, which may not be parenting-specific but clearly shape parental performance under specific conditions. Resolving these questions will require longitudinal and cell-type-specific recordings across behavioural contexts, combined with improved activity-labelling approaches that can directly test whether the same neurons are reused across parenting, mating and aggression.

A second major challenge is understanding how parental states are translated into specific actions. Considerable progress has been made in identifying hypothalamic and limbic nodes that promote or suppress caregiving, but far less is known about how these states are converted into motor programmes—such as retrieval, crouching, nest building, licking, defence or attack—through downstream midbrain and brainstem circuits. As recently shown for mating, these transformations may rely on downstream gating mechanisms that integrate hypothalamic state signals with sensory inputs to permit specific motor actions^62^. Determining these output pathways will be essential for understanding how hypothalamic state signals are converted into adaptive behavioural sequences.

The neural basis of paternal behaviour remains far less well understood than that of motherhood, particularly the post-mating switch from infanticide to care and the hormonal and circuit changes associated with fatherhood. Likewise, we know surprisingly little about how parental circuitry differs across species with uniparental, biparental or cooperative breeding systems. Do these species rely on modulation of a conserved circuit architecture, or do they differ in more fundamental aspects of functional connectivity and circuit composition? Work in voles and oldfield mice suggests that species differences in parental strategy can arise through differential neuromodulation of a shared circuit architecture rather than fundamentally distinct circuitry^236,237^, but whether this generalises across mammals remains unclear. Finally, understanding what fails when parental behaviour is disrupted — for example in postpartum psychiatric disorders — remains an important translational challenge. Defining how parental circuits become unstable or maladaptive may provide broader insight into how social behaviour breaks down in disease.

Addressing these questions will require both technical and conceptual advances. In established model systems, especially mice, increasingly powerful tools for longitudinal and cell-type-specific neural recording, improved genetic access to developmentally defined circuit elements, and better approaches to monitor and manipulate neural plasticity should make it possible to track how parental circuits emerge and change over time. At the same time, studying parenting in more naturalistic conditions will be crucial, as many key features of caregiving — including social structure, offspring demand, ecological constraints and competing motivational states — are difficult to capture in simplified laboratory assays. Comparative work in emerging model systems, particularly species that exhibit cooperative breeding, will be equally important for establishing conserved principles of parental circuit organisation^80,236,238^.

Parenting has long served as a powerful model for studying instinctive behaviour. It is now becoming equally valuable for understanding how neural circuits balance stability with flexibility, and how they are remodelled to meet ever-changing behavioural demands across physiological states and throughout life. Understanding the parental brain will therefore likely reveal broader principles by which the nervous system generates adaptive social behaviour.

## Box 1 Mammalian parenting strategies

Sex differences in mammalian parental care emerge from asymmetries in the reproductive investment of males and females and from ecological conditions that shape the relative fitness payoffs of mating versus caregiving^2^. Because mammalian females necessarily invest in gestation and lactation, their minimum required parental investment is typically higher than that of males, biasing the balance toward maternal care^2,3^. Biparental care or cooperative breeding evolves when the involvement of additional caregivers substantially increase offspring survival and when the costs of care are reduced. In the case of alloparental care, this occurs when the marginal fitness benefits of additional care are high, such as when offspring are numerous, highly dependent or costly to provision, carry or protect, and when the costs to caregivers are sufficiently low or offset by direct or indirect fitness gains. Both biparental care and cooperative breeding are often associated with **social monogamy**, which tends to evolve when breeding females are spatially dispersed and intolerant of one another, limiting a male’s ability to monopolise multiple mates; under these conditions, guarding a single female becomes an efficient strategy^23,25^.

Biparental or paternal care (**Box 2**) often follows the evolution of social monogamy, consistent with the idea that sustained male–female association creates conditions in which male care becomes advantageous^23^. Prominent examples of this type of care include California mice (*Peromyscus californicus*), which demonstrate biparental care, and prairie voles (*Microtus ochrogaster*), which exhibit substantial paternal care compared with uniparental rats and mice^244,245^. Recent work in monogamous oldfield mice (*Peromyscus polionotus* has demonstrated one mechanism involved in the evolution of socially monogamous parental behaviour. Oldfield mice have evolved a novel adrenal cell type that produces 20α-hydroxyprogesterone, a progesterone metabolite that promotes parental care after conversion to allo-diol in the brain, where it acts as a GABA_A_ receptor antagonist^236^.

Cooperative breeding — in which offspring are routinely cared for by individuals in addition to their parents — has evolved in a small number of socially monogamous species that regularly produce multiple offspring per litter, including meerkats (*Suricata suricatta*), marmosets (*Callithrix jacchus*) and tamarins (*Saguinus oedipus*)^27,246–248^. In these species, the combination of stable pair bonds and high offspring demand likely increases the marginal benefit of additional caregivers: multiple dependent offspring substantially raise provisioning and carrying demands, while the existence of a stable breeding pair can promote delayed dispersal of older offspring, creating opportunities for helping behaviour^27^. In such species, multiple group members contribute to protecting, warming, feeding and/or carrying infants, and these contributions are associated with enhanced offspring fitness^246,247,249^.

## Box 2 Paternal care

Paternal care provides an opportunity to understand how parenting circuits can be recruited independently of pregnancy. Although rare across mammals, comparative phylogenetic analyses suggest that biparental care has evolved repeatedly from female-only care^250^. At the same time, paternal care often co-occurs with pair bonding, territorial defence and changes in social status (for example, becoming a resident, pair-bonded breeding male), complicating efforts to distinguish mechanisms specific to caregiving^20,251^. Fathers in biparental species perform many of the same non-lactational pup-care behaviours as mothers^252,253^, including retrieving, grooming and huddling over pups^252,253^. Within *Peromyscus*, paternal care is observed in monogamous oldfield and California mice, but is largely absent from promiscuous eastern deer mice (*Peromyscus maniculatus*) and white-footed mice (*Peromyscus leucopus*)^254,255^.

No pregnancy-like endocrine ‘on-switch’ for parental behaviour has been identified in males. Instead, studies in both wild and laboratory mice showed that ejaculation triggers a delayed post-mating switch, such that pup-directed attack is progressively suppressed and parental responses emerge around the time the male’s own offspring would be expected^240–243^. However, mating alone is not always sufficient. Cohabitation with a pregnant mate can suppress infanticide even before birth, whereas males housed with non-pregnant females remain far more likely to attack pups^256,257^. The post-mating switch is reversible, but experienced fathers show a more stable suppression of infant-directed aggression regardless of the female’s current reproductive state^256,257^. Thus, paternal behaviour is regulated by a combination of post-mating timing, social context and prior experience. The relative contributions of copulation, cohabitation and female reproductive state differ across genetic backgrounds^52,258,259^. Work in African striped mice (*Rhabdomys pumilio*) further shows that prior mating is not an absolute prerequisite for male care and that the MPOA expression level of the *Agouti* gene scales with group density and is negatively associated with parental behaviour^238^.

In mice, the male transition to caregiving seems to occur in two stages: from infanticide to pup tolerance, and from tolerance to active caregiving. Ejaculation appears sufficient to suppress aggression, whereas cohabitation with a pregnant female and subsequent pup exposure promote the emergence of full paternal behaviour^14,91^. Distinct forms of circuit plasticity seem to support these transitions. Mating and cohabitation reduce inhibition from cocaine- and amphetamine-regulated transcript (CART)-positive medial amygdala neurons onto central medial preoptic area (MPOA) neurons, whereas parental experience strengthens inhibitory transmission onto neurons in the rhomboid nucleus of the bed nucleus of the stria terminalis (BNSTrh), which drive pup-directed aggression in both males and females^14,91^. These findings support a model in which different nodes of the parenting circuit are sequentially remodelled to first suppress infanticide and then promote active care.

At the circuit level, paternal behaviour recruits much of the same architecture that supports maternal care. Pup-directed aggression strongly depends on vomeronasal and tactile pathways responsive to both pup-derived chemosignals and physical pup features^32,52^. As in females, MPOA lesions switch fathers from caregiving to infanticide, whereas BNSTrh lesions reduce infanticide in virgin males^79^. Similarly in naturally biparental California mice, MPOA lesions disrupt parental behaviour in both males and females^260,261^. Additional shared anti-parental pathways include amygdalohippocampal area (AHi)– MPOA projections, which promote aggression over caregiving, and UCN3-expressing perifornical area (PeFA) neurons, which drive infant-directed neglect and aggression^89,90^. A recent preprint has suggested that reciprocal MPOA–BNST dynamics are particularly important^91^. Recent work further identified a CART- and ESR1-expressing population of neurons in the principal BNST that drives infant-directed aggression: fathers show strengthened inhibitory synaptic input onto these neurons from the anterior commissure nucleus (ACN)^262^.

Hypothalamic oxytocin and arginine vasopressin (AVP) signalling play a central role in paternal caregiving. In male mice, oxytocin release from PVN neurons is required for paternal behaviour, and optogenetic activation of these neurons is sufficient to induce caregiving even in virgin males^17^. During fatherhood, excitatory input from the lateral hypothalamus onto oxytocin-expressing PVN neurons (PVN^*Oxt*^ neurons) is strengthened, enhancing oxytocin release and promoting caregiving^17^. AVP-expressing PVN neurons also promote caregiving, acting via AVP-sensitive oxytocin receptor signalling in the MPOA and ACN^263^. Similar mechanisms likely exist in other species as PVN^*Oxt*^ neurons are also activated during paternal behaviour and promote caregiving in male mandarin voles (*Lasiopodomys mandarinus*)^264^.

Endocrine and neuromodulatory influences on paternal care are generally subtler than in females. In California mice, castration reduces paternal care and pup grooming, whereas testosterone (T) replacement restores both^265^. This effect may involve local aromatase signalling within the MPOA, and transient T rises during courtship can predict later paternal behaviour^266,267^. In prairie voles, castration reduces paternal responsiveness and AVP-positive projections to the lateral septum (LS)^268^, although paternal care can remain relatively steroid-independent in other species, including Campbell’s dwarf hamsters (*Phodopus campbelli*)^269,270^. In humans, T typically declines across the transition to fatherhood and lower T is more consistently associated with caregiving and relationship investment than with mating effort^271,272^. In parallel, prolactin and oxytocin levels respond to infant cues and parent–infant contact in both humans and rodents^273–276^ and basal circulating prolactin is required for paternal responses in male mice^153^. Collectively, current evidence supports a model in which paternal care emerges primarily through state-dependent recruitment and sequential remodelling of a shared caregiving circuit rather than via male-specific pathways^47,85^.

## Glossary

Agonistic behaviour: Any social behaviour related to fighting, conflict, or competition, including threats, aggression, and submission.
Alloparenting: Care of offspring provided by individuals other than biological parents, including extended family or non-kin.
Valence: The positive or negative significance assigned to a stimulus, which biases behaviour towards approach or avoidance.
Cooperative breeding: A social system in which, in addition to the breeding parents, non-breeding group members consistently help care for offspring.
Core parental circuit: The conserved set of neural populations and pathways that organises infant-directed caregiving behaviour.
Distributed architecture: A mode of neural organization in which behaviour emerges from interactions across multiple brain regions rather than from a single control centre.
Gonadectomy: Surgical removal of the gonads (ovaries or testes), used experimentally to eliminate endogenous sex hormone production. The term *gonadectomised* refers to individuals that have undergone this procedure.
Hypophysectomised: Refers to individuals that have undergone surgical removal of the pituitary gland, resulting in loss of pituitary hormone secretion.
Internal state: The physiological or motivational condition of an animal, including hormonal, metabolic or affective factors, that shapes how sensory cues are processed and translated into behaviour.
Nulliparous: Refers to an individual that has never given birth. Commonly used to describe females without prior reproductive experience.
Observational learning: The acquisition of behaviour through observing another individual perform that behaviour.
Parturition: The process of giving birth, marking the transition from pregnancy to the postpartum state.
Peripartum: The period immediately before, during and after parturition.
Reproductive state: The current physiological status of an individual in relation to reproduction, for example virgin, pregnant, lactating or postpartum.
Sensitisation: The gradual emergence of parental behaviour in a non-parental animal following repeated exposure to infants.
Social monogamy: A social system in which one male and one female form a stable pair association, often associated with joint territory defence or offspring care.
Vomeronasal organ (VNO): A chemosensory organ that detects non-volatile social cues and contributes importantly to close-range evaluation of conspecifics.
